# Editorial: Balance-controlling mechanism and fall-prevention strategy

**DOI:** 10.3389/fneur.2024.1385917

**Published:** 2024-03-05

**Authors:** Christina Zong-Hao Ma, Ringo Tang-Long Zhu, Meizhen Huang, Winson Chiu-Chun Lee, Yonghong Yang, Chengqi He

**Affiliations:** ^1^Department of Biomedical Engineering, The Hong Kong Polytechnic University, Kowloon, Hong Kong SAR, China; ^2^Research Institute for Smart Ageing, The Hong Kong Polytechnic University, Kowloon, Hong Kong SAR, China; ^3^Department of Rehabilitation Sciences, The Hong Kong Polytechnic University, Kowloon, Hong Kong SAR, China; ^4^School of Mechanical, Materials, Mechatronic and Biomedical Engineering, University of Wollongong, Wollongong, NSW, Australia; ^5^Department of Rehabilitation Medicine, West China Hospital, Sichuan University, Chengdu, China; ^6^Key Laboratory of Rehabilitation Medicine, West China Hospital, Sichuan University, Chengdu, China

**Keywords:** balance, posture, falls, neurology, fall prevention, fall risk, rehabilitation

Falls and fall-related injuries and deaths burden society heavily. Balance and gait disorders are the primary cause of falls in older adults. Currently, evidence-based training regimens are still lacking for some populations with a specific balance disorder, which calls for high-quality interventional studies to facilitate clinical practices. In addition, tackling the challenges of fall prevention demands more in-depth investigations of balance-control mechanisms, which may facilitate the more sensitive assessment of balance impairment and possibly the earlier detection of fall risks. These mechanisms are also expected to provide insights for the earlier, more targeted, and more effective fall-prevention management. We are happy to have published 9 articles in this research topic that advance our understanding of the balance-control mechanisms (He et al.; Jiang et al.; Sato et al.; Xing et al.; Santos et al.; Caronni et al.) and the latest evidence-based fall-prevention management (Xing et al.; Winser et al.; Ho et al.; Elrod et al.).

## Probing the balance-control mechanisms

Increasing number of the existing clinical and laboratory tests have been validated for assessing the postural balance. This has facilitated the understanding of how patients' balance performance and fall risks following some neurological impairments are affected. Sato et al. have used the static posturography to evaluate balance during quiet standing in 30 inpatients, who received a radiofrequency ablation neurosurgery to treat the essential tremor. They observed that after the surgery with the tremor symptoms been reduced, the patients could not immediately readjust the center of pressure to the body midline. This potentially suggested the need of rehabilitation, for improving postural balance, in the perioperative period when surgically treating the individuals with essential tremors. Caronni et al. have examined the criterion validities of several clinical tests for balance and gait assessment as measures to differentiate future fall risks in individuals with a neurological disability. The Mini-Balance Evaluation System Test (Mini-BESTest) and the turning duration of the Timed Up and Go (TUG) test were found to be able to predict the participants' fall incidences. These tests have shown promising applications in the fall-risk assessments for the populations with neurological disabilities, although they may inadequately be able to distinguish the fall risks in community-dwelling older adults (1).

In this Research Topic, some studies have also applied advanced methods or have proposed new methods of analyzing the whole-body postural control. Jiang et al. have investigated how the suspensory strategy is affected by the different knee flexion angles in healthy young adults during quiet standing. On top of the conventional assessment of center-of-mass displacement, they have used the time-frequency analysis to evaluate the sensory input and used the sample entropy, one non-linear analysis method of quantifying postural regularity, to evaluate the motor output for maintaining standing balance. Santos et al. have proposed a new parameter to indicate the postural instability in individuals with the Parkinson's Disease (PD), based on the cost-effective motion capture of head movements in quiet standing and the use of movement element decomposition method. The parameter was found to differentiate the individuals among the early stages of PD progression better compared to several other clinical tests for balance performance.

Apart from the analysis of whole-body postural sways, recent studies have delved deeper into the roles of central nervous system and neuromuscular system in balance control. He et al. have used functional near-infrared spectroscopy to investigate the stroke survivors' cortical activation during walking. By comparing healthy walking, functional electrical stimulation (FES)-assisted walking, and non-FES walking, they have observed some asymmetric activation patterns in the investigated cortical areas for stroke survivors. Regarding the motor output pathway, recent studies have investigated the speed of multiple major lower-limb muscles' activation in maintaining reactive standing balance by analyzing the timing and rising rate of electromyographic (EMG) signals, revealing that ankle muscles have the faster response (2, 3). In addition to EMG, with the advancing of wearable technologies, some techniques, such as the ultrasound imaging of muscles, have been available to detect the muscle morphological changes in dynamic situations and assess balance performance (4, 5). Such muscular mechanisms provide new insights for improving balance or relieving the balance and gait disorders.

## Exploring the fall-prevention strategies

Since the causes of falls are multi-factorial, effective fall-prevention strategies are not confined to improve the balance and gait performance only (Xing et al.). Winser et al. have conducted a randomized controlled trial to examine the effectiveness and cost of integrated cognitive and balance training (CIBT) on balance and falls in individuals with cerebellar ataxia. The CIBT improved the limit of stability, a measure of volitional balance control, while it did not exhibit better effects on reducing falls compared to the conventional single-task training. Ho et al. have systematically reviewed the effectiveness of robotic-assisted upper-limb rehabilitation in individuals with cervical spinal cord injuries, since the upper-limb reach-and-grasp responses and the arm swings are also important for maintaining balance and avoiding falls. Elrod et al. have reported their case study on the development of academic-community partnerships for delivering fall-prevention programs in an American metropolitan setting. The programs were found successful in reaching the community-dwelling older adults with low to moderate fall risks but not for those at high risk. They have identified some key facilitators and barriers of pragmatic implementation, which may lend experience for the delivery of fall-prevention management in other areas.

In summary, the studies presented in this Research Topic provide updated insights into the clinical applications of balance assessment in specific populations, the state-of-art analysis methods of balance control, and the evidence on the effectiveness and actual implementation of specific fall-prevention programs. We expect that these efforts can facilitate current clinical practices in fall prevention and imply further research on probing balance-control mechanisms.

## Author contributions

CM: Writing—original draft, Writing—review & editing. RZ: Writing—original draft, Writing—review & editing. MH: Writing—review & editing. WL: Writing—review & editing. YY: Writing – review & editing. CH: Writing—review & editing.
